# Is reduction in appetite beneficial for body weight management in the context of overweight and obesity? Yes, according to the SATIN (Satiety Innovation) study

**DOI:** 10.1017/jns.2019.36

**Published:** 2019-11-27

**Authors:** Thea Toft Hansen, Bethan R. Mead, Jesús Francisco García-Gavilán, Sanne Kellebjerg Korndal, Joanne A. Harrold, Lucia Camacho-Barcía, Christian Ritz, Paul Christiansen, Jordi Salas-Salvadó, Mads Fiil Hjorth, John Blundell, Mònica Bulló, Jason C. G. Halford, Anders Sjödin

**Affiliations:** 1 Department of Nutrition, Exercise and Sports, Section for Obesity Research, Faculty of Science, University of Copenhagen, 1958 Copenhagen, Denmark; 2 Department of Psychological Sciences, University of Liverpool, Liverpool L69 7ZA, UK; 3 Human Nutrition Unit, Faculty of Medicine and Health Sciences, Institut d'Investigació Sanitària Pere Virgili, Rovira i Virgili University, 43201 Reus, Spain; 4 CIBER Fisiopatología de la Obesidad y Nutrición (CIBEROBN), Instituto de Salud Carlos III, 28029 Madrid, Spain; 5 Institute of Psychological Sciences, Faculty of Medicine and Health, University of Leeds, Leeds LS2 9JT, UK

**Keywords:** Satiety, Hunger, Food innovation, Weight loss, Weight maintenance, E %, energy percentage, LED, low-energy diet, PYY, peptide YY, SATIN, Satiety Innovation, TFEQ, three-factor eating questionnaire, VAS, visual analogue scale

## Abstract

New dietary-based concepts are needed for treatment and effective prevention of overweight and obesity. The primary objective was to investigate if reduction in appetite is associated with improved weight loss maintenance. This cohort study was nested within the European Commission project Satiety Innovation (SATIN). Participants achieving ≥8% weight loss during an initial 8-week low-energy formula diet were included in a 12-week randomised double-blind parallel weight loss maintenance intervention. The intervention included food products designed to reduce appetite or matching controls along with instructions to follow national dietary guidelines. Appetite was assessed by *ad libitum* energy intake and self-reported appetite evaluations using visual analogue scales during standardised appetite probe days. These were evaluated at the first day of the maintenance period compared with baseline (acute effects after a single exposure of intervention products) and post-maintenance compared with baseline (sustained effects after repeated exposures of intervention products) regardless of randomisation. A total of 181 participants (forty-seven men and 134 women) completed the study. Sustained reduction in 24-h energy intake was associated with improved weight loss maintenance (*R* 0·37; *P* = 0·001), whereas the association was not found acutely (*P* = 0·91). Suppression in self-reported appetite was associated with improved weight loss maintenance both acutely (*R* −0·32; *P* = 0·033) and sustained (*R* −0·33; *P* = 0·042). Reduction in appetite seems to be associated with improved body weight management, making appetite-reducing food products an interesting strategy for dietary-based concepts.

Increased prevalence of obesity and co-morbid lifestyle diseases places a great burden on society and individuals^(1)^. Even a modest weight loss of 5–10 % of the initial body weight has been shown to result in beneficial effects on cardiometabolic risk factors^(2,3)^. However, weight loss programmes designed to create a period of negative energy balance are often followed by weight regain^(4,5)^. Surgical treatments of obesity seem more proficient in obtaining sustained weight losses^(6–8)^, but non-invasive and less costly concepts are needed for treatment and prevention of overweight and obesity, including prevention of weight regain. Thus, new dietary-based treatments for body weight management, particularly after weight loss, are needed.

One target could be to trigger physiological mechanisms that reduce or delay appetite, leading to reduction in energy intake. Energy intake is to a great extent controlled by the individual's appetite, which is affected by a plethora of physiological, psychological and sociological factors^(9)^. The physiological and psychological factors that affect appetite are highly integrated and both are affected by signals from peripheral organs^(10)^. Perceived satiety and hunger may therefore predict the individual's ability to manage their body weight^(11)^, making an increased appetite a considerable cause for failed weight loss and weight loss maintenance^(10,12,13)^. It has been shown that diet-induced weight loss increases systemic concentrations of the orexigenic hormone ghrelin as well as decreases in anorexigenic hormones like glucagon-like peptide-1 and peptide YY^(14–16)^ simultaneously as energy expenditure is typically reduced^(17,18)^. Thus, it seems reasonable to assume that these counteracting mechanisms can limit weight loss and be important for the failed weight loss maintenance typically seen even after very successful weight losses^(4)^. Interestingly, surgically induced weight losses seem to counteract these physiological mechanisms and even decrease the appetite, also after a major weight loss^(19,20)^. The hypothesis that appetite affects body weight management thereby seems plausible.

This is to some extent supported in our recently published systematic review and meta-analysis^(21)^; however, most of the currently available literature is not optimally designed to investigate this hypothesis. Previous studies examining associations between individual changes in appetite and body weight changes are very limited. We were only able to identify two studies, and these found that decreased *ad libitum* energy intake was associated with subsequent weight loss^(22,23)^. Thus, to our knowledge, it has never been assessed if reduction in appetite is beneficial for weight loss maintenance.

In a similar way as the previous studies, data from a large intervention study were used to investigate if reduction in appetite also can be shown to be associated with improved weight loss maintenance. Appetite was assessed before and after a weight loss maintenance period by *ad libitum* energy intake and self-reported appetite evaluations and related to body weight changes during the weight loss maintenance period. More specifically, we evaluated if individual variations in 1-d appetite assessments were associated with weight loss maintenance. Based on this, we aimed to evaluate if reduction in these appetite assessments can be seen as markers of a beneficial physiological effect on body weight management in the context of overweight and obesity. The primary objective was to investigate if reduction in appetite is associated with improved weight loss maintenance. Secondarily, we investigated if a high level of appetite after weight loss is a risk factor for weight regain.

## Methods

**Fig. 1.**
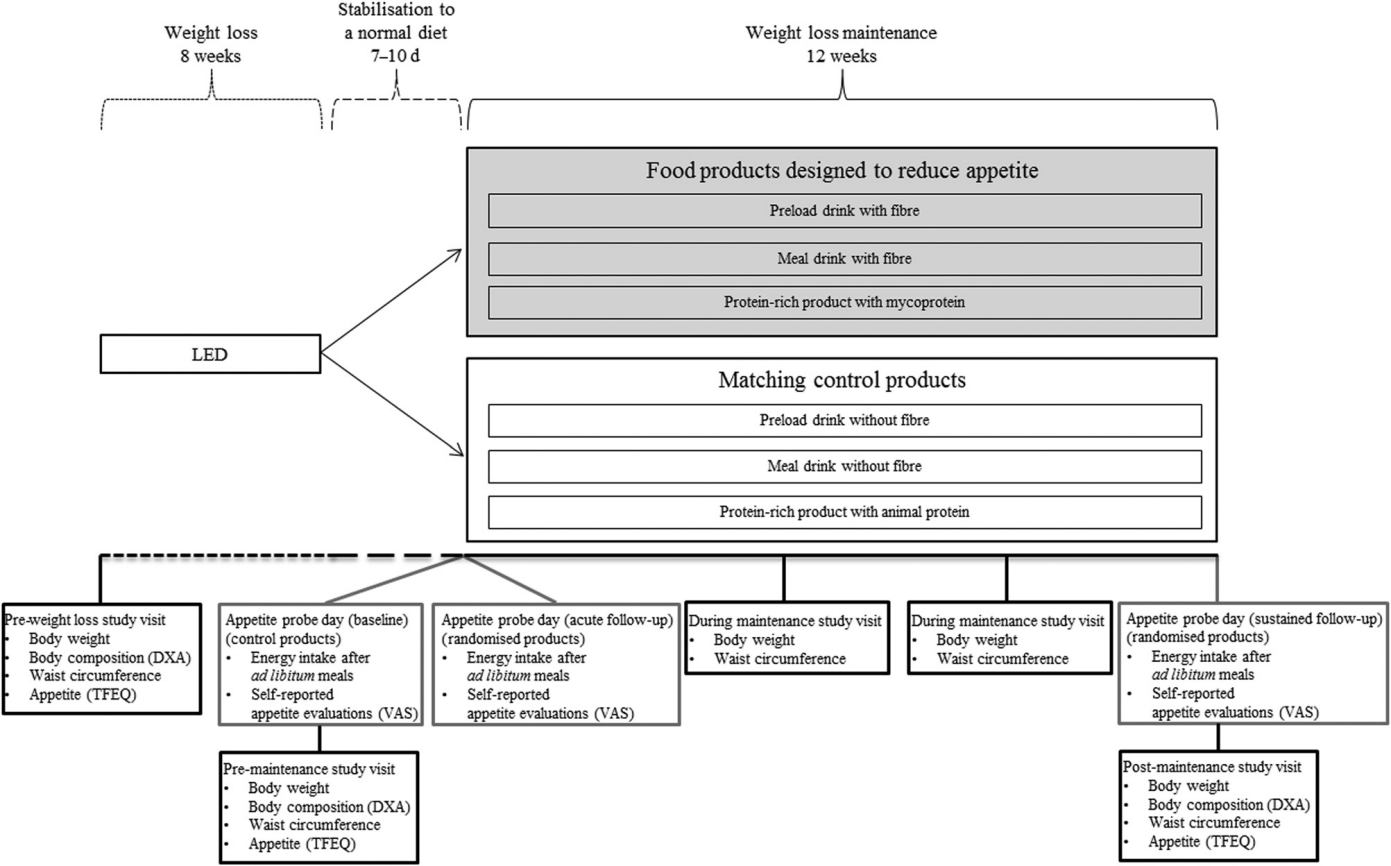
Overview of the study design and data collection involved in the eight visits assessing appetite and body weight. Chronologically in the order the participants completed the low-energy diet (LED) period at each of the study sites, participants were randomised to receive one of six different products based on the following stratification: site (Copenhagen/Reus/Liverpool), sex (male/female), age (20–42 years/43–65 years) and relative weight loss achieved during the 8-week LED period (<10 %/≥10 %). On the baseline appetite probe day, control products corresponding to the type of product which the participants were allocated to were used for all participants. For the following two appetite probe days, the products which the participants were allocated to during the intervention period were used. DXA, dual-energy X-ray absorptiometry; TFEQ, three-factor eating questionnaire; VAS, visual analogue scale.

This cohort study was nested within the European Commission project Satiety Innovation (SATIN) work package 5 and primarily includes data collected during the weight loss maintenance period. The SATIN work package 5 study was conducted as a multi-centre study including participants from Denmark (Copenhagen), Spain (Reus) and England (Liverpool). The weight loss maintenance period followed an initial 8-week low-energy diet (LED) period. During the LED period, men were assigned to consume 5020 kJ/d and women 4184 kJ/d from the Modifast^®^ (Nutrition et Santé SAS) formula diet, and the participants met every second week at group sessions for provision of products and support from each other and a dietitian. The LED period is described in more detail elsewhere^(24)^. Participants achieving ≥8 % weight loss during the LED^(25)^ were included in the subsequent 12-week randomised double-blind parallel intervention with food products designed to reduce appetite or matching control products (Fig. 1).

### Study participants

Prior to screening, potential participants were informed about the entire study design as well as the exclusion after the LED period if achieving <8 % weight loss. Screening identifying eligibility with inclusion and exclusion criteria was controlled before inclusion and initiation of the LED period. Men and women with overweight or obesity but otherwise healthy were recruited for the study based on advertisement through different media sources. Participants met inclusion criteria if they were aged between 20 and 65 years, had a BMI of ≥27·0 and ≤35·0 kg/m^2^ and a fat mass of ≥23 % (assessed by bioimpedance).

Exclusion criteria included contradictions related to the use of LED products or the study products used during the maintenance period (including dislike of the products), known diseases which may affect energy expenditure and/or appetite, dietary patterns interfering with the study protocol (e.g. vegetarians who would be unable to consume the standardised meals at the appetite probe days during the weight loss maintenance period), body weight changes ±3 kg within the past 3 months prior to inclusion, engagement in strenuous exercise ≥5 h/week, smoking or smoking cessation within the past 3 months prior to inclusion and/or nicotine use (including electronic cigarettes) and specific diseases (detailed described elsewhere^(24)^). Participants achieving <8 % weight loss during the LED period were discontinued after the LED.

The study was conducted in accordance with Good Clinical Practice and the study protocol and study forms complied with the relevant sections of the Declaration of Helsinki. Participants received written and oral information about the study and written informed consent was obtained before any study-related procedures were initiated. Recruitment and testing took place at the Department of Nutrition, Exercise and Sports, University of Copenhagen, Denmark between January and November 2016, at the Department of Biochemistry and Biotechnology, University Rovira i Virgili; Institute of Health IISPV and The Spanish Biomedical Research Centre in Physiopathology of Obesity and Nutrition (CIBERobn), ISCIII between January and December 2016 and at the Department of Psychological Sciences, Institute of Psychology, Health and Society, University of Liverpool between April 2016 and July 2017. The study was approved by the Municipal Ethical Committee of Copenhagen/Scientific Ethics Committee of the Metropolitan regions of Denmark (journal no. H-15008553), the Danish Data Protection Agency (journal no. 2015-57-0117) and the Clinic Investigation Ethical Committee of the Hospital University Sant Joan de Reus (journal no. 15-07-30/7assN2). Sponsorship was obtained from the University of Liverpool (reference UoL001153) and related ethical approval was received from Preston NRES Committee North West (health research authority) (journal no. 16/NW/0135). The study was registered at www.ClinicalTrials.gov (ID NCT02485743). All study procedures were aligned between sites before initiation of the study and on-site monitoring visits were carried out by an independent monitor.

### Study visits

The present study comprised of a screening visit and additional sixteen visits to the research facilities. The present paper includes data from eight of these visits (Fig. 1). Participants who achieved ≥8 % weight loss during the LED were randomly assigned to receive either products designed to reduce appetite or matching controls during the weight loss maintenance period. Chronologically in the order the participants completed the LED period at each of the study sites, participants were randomised in a 1:1 ratio to receive one of six different products based on the following stratification: site (Copenhagen/Reus/Liverpool), sex (male/female), age (20–42 years/43–65 years) and relative weight loss achieved during the 8-week LED period (<10 %/≥10 %). The products were selected based on previously established evidence for an ability to reduce appetite compared with their matching control product^(26–30)^. The control products matched the intervention products in terms of visual appearance, taste, etc. The products were labelled with three-digit codes according to the type of product. One designated independent person at the University of Copenhagen was responsible for allocation of codes for each of the six test products and distribution of codes directly to the product providers. Due to visual differences between the three types of products and different instructions for consumption, the study personnel and participants were aware of the type of product, but not whether the product was designed to have enhanced appetite-reducing effects or not. Treatment allocation was retained until data analyses were completed. Compliance was evaluated and enforced based on self-reports in diaries that the participants handed in at the study visits during the maintenance period. The products consisted of: a preload-drink with or without an inulin-based soluble fermentable fibre and extracts of yerba matè, guarana and damiana to be consumed 10 min before breakfast and lunch; a meal drink with or without α-galacto-oligosaccharides and soluble fibre extracted and purified from field peas (*Pisum sativum*) to be consumed during breakfast and as an afternoon snack; a protein-rich product of mycoprotein with regular animal protein as control provided in a selection of five varieties to be consumed as one serving per d five times per week. Since the aim of the analyses reported within the present study is not dependent on the effects of the specific products, specifications of the products and potential specific effects on appetite and body weight will not be described in more detail. During the weight loss maintenance period, the participants were additionally instructed to follow respective national dietary guidelines. To avoid potential effects on appetite of an ongoing negative energy balance, 7–10 d of stabilisation to a normal diet complying with respective national dietary guidelines were required before any study procedures related to the weight loss maintenance period were carried out. Compliance with national guidelines was checked by verbal reporting and enforced at visits with a dietitian. In relation to the weight loss maintenance period, a total of three appetite probe days were performed. Before initiating the weight loss maintenance period, a first appetite probe day assessing baseline appetite was performed in connection with pre-maintenance study visits assessing body weight, etc. Within 1 week after the baseline appetite probe day and again upon completion of the weight loss maintenance period, a second and a third appetite probe day were performed and post-maintenance study visit assessing body weight, etc. were performed along with the third appetite probe day. Appetite probe day and associated study visit were performed a maximum of 3 d apart. Every fourth week during the maintenance period (i.e. two visits), the participants consulted the research facilities for measurement of body weight and waist circumference as well as for consultations with a dietitian (Fig. 1).

### Assessments of appetite

Appetite was assessed at appetite probe days by objectively measuring 24-h energy intake (all energy consumed at the appetite probe days: this comprised a standardised fixed breakfast as well as *ad libitum* lunch, dinner and snack box), energy intake from *ad libitum* meals as well as by self-reported appetite evaluations (visual analogue scales; VAS). At arrival after an overnight fast and using the least strenuous means of transportation, the 8 h appetite probe days comprised a standardised fixed breakfast meal (providing 2000 kJ corresponding to approximately 20 % of the daily energy requirement for an average adult^(31)^) as well as an *ad libitum* lunch and *ad libitum* dinner. Additionally, an *ad libitum* snack box was provided to take home for the remainder of the day and potential left-overs as well as diaries of any additional foods and energy-containing beverages were handed in on the following day for the assessment of energy intake for the reminder of the appetite probe day. The content and timing of the meals varied between study sites, representing local eating habits. The *ad libitum* meals served at the research facilities (i.e. lunch and dinner) were homogeneous and all meals were designed to have equal macronutrient distribution between sites (15% energy (E %) protein, 55 E % carbohydrate, 30 E % fat (maximum 0·5 E % deviations)) and were identical at all probe days at each site, i.e. no within-participant variation. The study products were included during the appetite probe days as instructed to be consumed during the intervention period. On the baseline appetite probe day, control products corresponding to the type of product which the participants were allocated to were used for all participants. This was followed by a second appetite probe day on the first day of the weight loss maintenance period in order to assess acute effects after a single exposure. A third appetite probe day was performed post-maintenance compared with baseline assessing sustained effects after repeated exposures. At the second and the third appetite probe days, the products which the participants were allocated to during the intervention period were used. Self-reported appetite evaluations were assessed using electronic VAS (Lenovo^®^ thinkpad^®^ tablet 10; Evascale, build by Jakob Lund Laugesen, University of Copenhagen) (used in Copenhagen) or pen-and-paper VAS (used in Reus and Liverpool) of 100 mm assessing feelings of satiety, fullness, hunger, desire to eat and prospective food consumption^(32–34)^ were applied just before and after each eating occasion as well as every 1 h over the course of the appetite probe days. An overall appetite suppression score for each time point was calculated by the equation: (satiety + fullness + (100 – hunger) + (100 – desire to eat) + (100 – prospective food consumption))/5, with 0 indicating higher appetite/less satiety and 100 indicating lower appetite/more satiety^(35)^. During the appetite probe days, the participants were seated one or two per room, and they were instructed to be focused on their eating and not to talk to each other during the *ad libitum* meals and when answering VAS.

Additionally for explanatory analyses, eating behaviour was assessed by the validated three-factor eating questionnaire (TFEQ) and used as an indication of appetite. The TFEQ includes fifty-one questions related to appetite and eating behaviour generating scores of: restraint (cognitive control of the frequency, amount and types of food being eaten); disinhibition (lack of control over eating behaviour in spite of conscious awareness hereof); and hunger (susceptibility to hunger)^(36)^. The minimum to maximum score is 0–21, 0–16 and 0–14 for restraint, disinhibition and hunger, respectively^(36)^. The participants completed the questionnaire pre-weight loss, pre-maintenance and post-maintenance. For practical reasons, the participants could complete the questionnaires at home right before or after the study visits. A standard front page, layout and introduction text were attached and the questions were always presented in the same order as in the validated version. Other psychological variables measured throughout the study will be analysed in more detail in a forthcoming paper.

Measures of appetite were analysed in the pre-planned order specified in Table 1, including the primary endpoint used as markers of altered appetite for each of the objectives as well as additional endpoints used for explanatory analyses.

**Table 1.** Pre-planned order in which measures of appetite were analysed separated by the objectives

Primary objective: Is reduction in appetite associated with improved weight loss maintenance?	24-h energy intake including all energy consumed on the appetite probe days (standardised fixed breakfast as well as *ad libitum* lunch, dinner and snack box) (primary endpoint) Accumulated energy intake from the *ad libitum* lunch and dinner Energy intake from each of the *ad libitum* meals: lunch, dinner, snack box Overall appetite suppression VAS score* Each of the VAS scores related to decreased appetite: satiety, fullness Changes in TFEQ restraint, disinhibition and hunger during the weight loss maintenance period
Secondary objective: Is a high level of appetite after weight loss a risk factor for weight regain?	Pre-maintenance VAS scores related to increased appetite: hunger, desire to eat, prospective food consumption (primary endpoint) Changes in TFEQ restraint, disinhibition and hunger during the LED Pre-maintenance TFEQ restraint, disinhibition and hunger

VAS, visual analogue scale; TFEQ, three-factor eating questionnaire; LED, low-energy diet.

* Overall appetite suppression score = (satiety + fullness + (100 – hunger) + (100 – desire to eat) + (100 – prospective food consumption))/5; 0 indicates higher appetite/less satiety and 100 indicates lower appetite/more satiety. The self-reported appetite evaluations (VAS) were summarised as incremental AUC (trapezoidal rule).

### Anthropometric measurements

Body weight while wearing light clothing and having emptied the bladder was measured to the nearest 0·1 kg on calibrated scales (Copenhagen: Lindell Tronic 8000; Reus: Tanita SC-331S, Tanita Corporation of America Inc.; Liverpool: Seca 799 Electronic Column Scales Class (III)) in a fasting condition at the pre-weight loss, pre-maintenance and post-maintenance study visits and in a non-fasting condition at the study visits during the intervention period. Height without shoes was measured to the nearest 0·5 cm using wall-mounted stadiometers (Copenhagen and Reus: Seca; Liverpool: Seca 220 Telescopic Measuring Rod). BMI was calculated with the formula: body weight (kg)/(height(m))^2^. Body composition measured at the pre-weight loss, pre-maintenance and post-maintenance study visits was determined by dual-energy X-ray absorptiometry (DXA) (GE Lunar iDXA™ and enCORE software, version 16.2 (Copenhagen and Liverpool); version 13.4 (Reus)). Waist circumference while wearing light clothing was measured to the nearest 0·5 cm using a non-elastic tape measure. The waist circumference was measured at the midpoint between the bottom of the rib cage (last floating rib) and the top of the iliac crest with the measuring tape around the trunk in a horizontal plane. Once the measuring tape was placed, the participants were asked to relax with both arms at his/her side and to breathe normally in order to take each measurement on the exhales^(37)^.

### Calculations of associations between appetite and body weight

The following associations were examined to address the primary objective:

*Acute effects on appetite after a single exposure*:

- Difference in measures of appetite (*ad libitum* energy intake and VAS) between the first and the second appetite probe days *v.* difference in body weight between the pre-maintenance and the post-maintenance study visits using data from all participants regardless of randomisation.

*Sustained effects on appetite after repeated exposures*:

- Difference in measures of appetite (*ad libitum* energy intake and VAS) between the first and the third appetite probe days *v.* difference in body weight between the pre-maintenance and the post-maintenance study visits using data from all participants regardless of randomisation.
- Difference in TFEQ scores between the pre-maintenance and the post-maintenance study visits *v.* difference in body weight between the pre-maintenance and the post-maintenance study visits using data from all participants regardless of randomisation.

The following associations were examined to address the secondary objective:

- Pre-maintenance VAS scores indicating a high level of appetite assessed at the first appetite probe day *v.* difference in body weight between the pre-maintenance and the post-maintenance study visits using data from all participants regardless of randomisation.
- Difference in TFEQ scores between the pre-weight loss and the pre-maintenance study visits *v.* difference in body weight between the pre-maintenance and the post-maintenance study visits using data from all participants regardless of randomisation.
- Pre-maintenance TFEQ scores *v.* difference in body weight between the pre-maintenance and the post-maintenance study visits using data from all participants regardless of randomisation.

### Statistical analyses

The study was designed to have 120 participants (total number of subjects envisioned to complete the three arms of products designed to reduce appetite) completing the study in order to have above 90 % power to detect an association between reduction in appetite and body weight changes based on the assumption of an expected mean reduction in *ad libitum* energy intake of 400 kJ with an sd of 800 kJ and a corresponding mean weight regain of 1·5 kg with an sd of 4·7 kg.

Baseline characteristics were summarised using means and standard deviations. For all outcomes, linear mixed models were fitted with site included as a random effect. Since overall changes in body weight and waist circumference were based on several measurements from the maintenance period, participant further needed to be included as a random effect in these models. All models included the following fixed effects: age, sex, pre-maintenance body weight or BMI, body weight change during the LED period and baseline measure of appetite of interest (e.g. when examining association between differences in 24-h energy intake and changes in body weight, the model was adjusted for 24-h energy intake at baseline). The self-reported appetite evaluations were summarised as incremental AUC (trapezoidal rule). For all models, assumptions of normality and homogeneity of variance were assessed through visual inspection of histograms and quantile−quantile plots and plots of residuals against the fitted values. Results are shown as estimated means with 95 % CI. Statistical analyses were carried out for complete-case data using Stata/SE 15.1 (StataCorp). A significance level of 0·05 was used.

## Results

### Study population

**Fig. 2.**
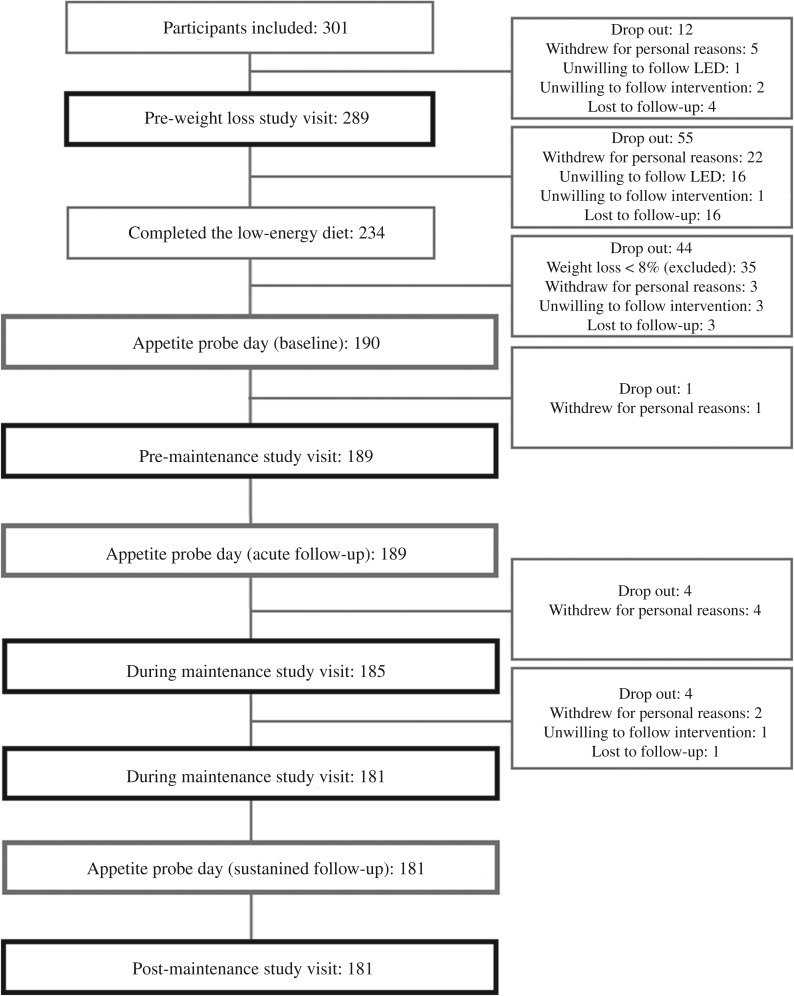
Flow chart of participants completing each visit including explanations for drop out. LED, low-energy diet.

From the total of 374 participants who responded to the advertisements, 301 participants were eligible for inclusion, but twelve dropped out prior to the pre-weight loss study visit. During the LED or immediately before initiating the weight loss maintenance period, an additional 100 participants dropped out or were excluded due to weight loss <8 %. Thereby, 189 participants (forty-eight men and 141 women) initiated the weight loss maintenance period (Fig. 2). Mean age was 47·8 (sd 9·7; range 20–65 years) years with body weight of 78·9 (sd 9·4) kg and BMI of 27·7 (sd 2·1) kg/m^2^ when initiating the weight loss maintenance period. No emergencies occurred whereby the study remained double-blinded throughout the course of the study.

### Overall weight loss maintenance

The participants completing the weight loss maintenance period (*n* 181) achieved a mean weight loss of 10·8 (95 % CI 10·5, 11·0) % after 8 weeks of the LED. After the 12-week weight loss maintenance period, the participants regained only 0·6 (95 % CI 0·2, 1·1; range −9·4 to 10·0) %. Changes in weight loss maintenance did not vary between men (*n* 47) and women (*n* 134) (χ^2^ = 4·21; *P* = 0·24). Overall pre-weight loss, pre-maintenance and post-maintenance assessments along with changes from pre-maintenance to post-maintenance are shown in Table 2 for all participants completing the weight loss maintenance period.

**Table 2.** Anthropometrics pre-weight loss, pre-maintenance and post-maintenance with changes during the weight loss maintenance period for all participants completing the weight loss maintenance period (*n* 181) (Mean values and standard deviations; mean changes and 95% confidence intervals)

	Pre-weight loss		Pre-maintenance		Post-maintenance		Changes after 12 weeks’ maintenance†	
	Mean	sd	Mean	sd	Mean	sd	Mean	95 % CI
Age (years)	47·9	9·6						
Anthropometry								
Body weight (kg)	88·4	10·9	79·0	9·5	79·5	10·0	0·5***	0·2, 0·8
BMI (kg/m^2^)	31·0	2·1	27·8	2·1	27·9	2·3	0·2	−0·3, 0·6
Fat mass (DXA, kg)	36·2	5·6	28·8	5·8	28·8	6·2	−0·1	−0·9, 0·7
Fat-free mass (DXA, kg)	52·2	9·3	50·2	9·0	50·7	9·0	0·5	−0·5, 1·5
Waist circumference (cm)	101·4	9·1	91·4	8·5	92·1	8·8	0·6	−0·1, 1·3

DXA, dual-energy X-ray absorptiometry.

*** Significant change from pre-maintenance to post-maintenance (*P* < 0·001).

† Changes in all measurements from the pre-maintenance to post-maintenance study visit were analysed by linear mixed models including adjustment for visit, age and pre-maintenance BMI (fixed effects) as well as participant and site (random effects).

### Relationship between reduction in appetite and weight loss maintenance

**Fig. 3.**
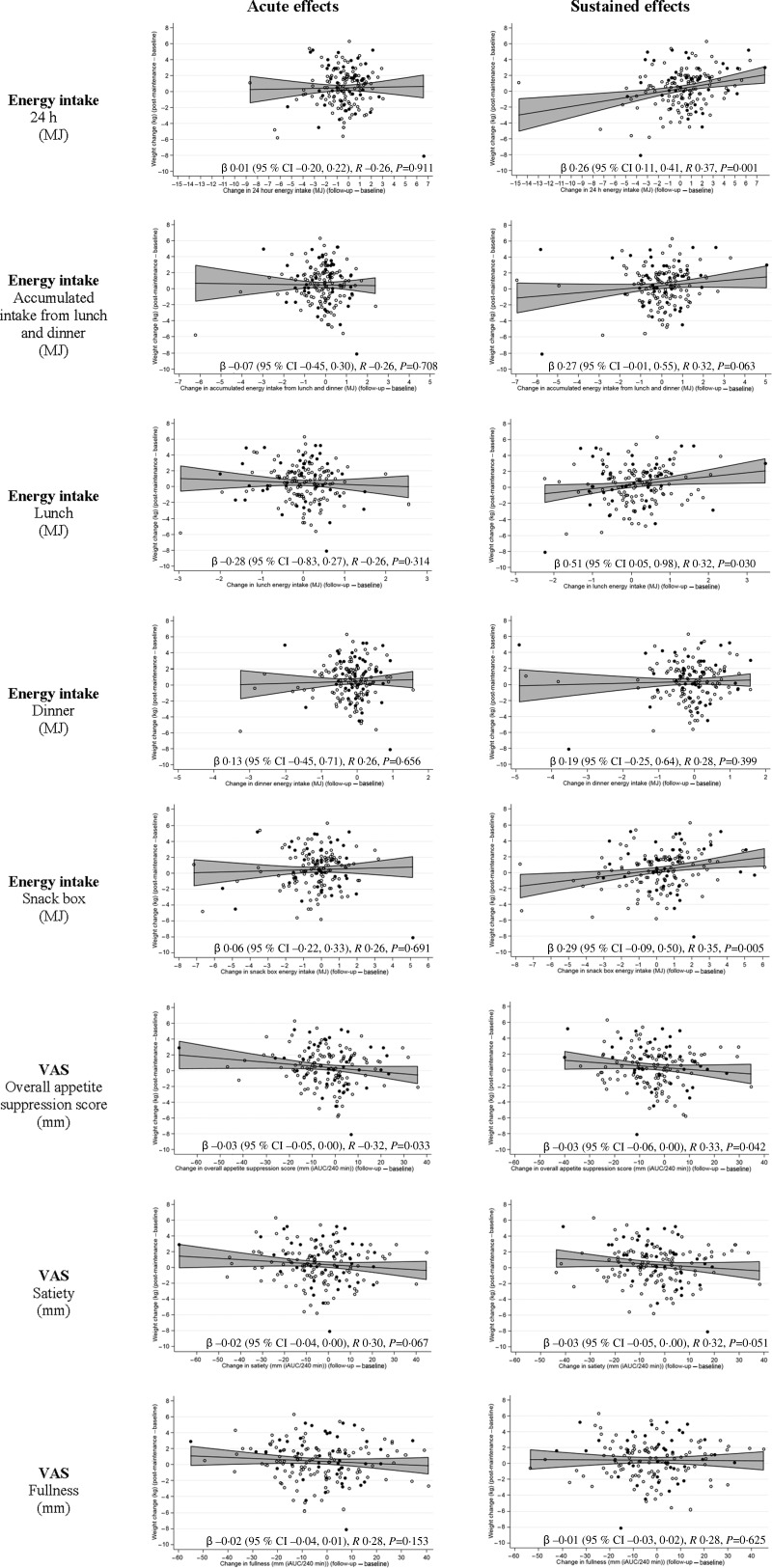
Relationship between changes in appetite (24-h energy intake, energy intake at each of the *ad libitum* meals and summarised incremental AUC (iAUC) of each of the self-reported appetite evaluations divided by acute (after a single exposure) and sustained (after repeated exposures) effects) and changes in body weight (kg) from pre- to post-maintenance. VAS, visual analogue scale; acute effects, difference in measures of appetite between the first and the second appetite probe days; sustained effects, difference in measures of appetite between the first and the third appetite probe days; overall appetite suppression score = (satiety + fullness + (100 – hunger) + (100 – desire to eat) + (100 – prospective food consumption))/5; 0 indicates higher appetite/less satiety and 100 indicates lower appetite/more satiety. Positive change in energy intake equals increased energy intake/higher appetite. Positive change in self-reported appetite evaluation equals decreased appetite. Positive weight change equals weight regain after the weight loss maintenance period. Data are presented as unstandardised regression coefficients (β) and 95 % confidence intervals and correlation coefficients using linear mixed models including adjustment for age, sex, pre-maintenance body weight, body weight change during the low-energy diet period and baseline measure of appetite of interest (e.g. when examining association between changes in 24-h energy intake and changes in body weight, the model was adjusted for 24-h energy intake at baseline) (fixed effects) as well as site (random effect). ●, Men (*n* 47); ○, women (*n* 134); ––, fitted line; 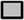, 95% CI.

Sustained reduction in 24-h energy intake was associated with improved weight loss maintenance (*R* 0·37; *P* = 0·001), mainly reflected in the energy intake from the *ad libitum* lunch (*R* 0·32; *P* = 0·030) and snack box (*R* 0·35; *P* = 0·005) (Fig. 3). The relationship between reduction in appetite and weight loss maintenance was further supported when appetite was expressed as overall appetite suppression score (*R* −0·33; *P* = 0·042) (Fig. 3). However, no relationship between acute changes in energy intake and weight loss maintenance was found (all *P* > 0·31) (Fig. 3), while acute enhancements in overall appetite suppression score were associated with improved weight loss maintenance (*R* −0·32; *P* = 0·033) (Fig. 3). Comparable results were found when examining associations between these measures of appetite and changes in fat mass during the weight loss maintenance period (see Supplementary Fig. S1). Sustained reductions in 24-h energy intake were found to explain 22 % of the variation in fat mass change during the weight loss maintenance period (*P* < 0·001) (see Supplementary Fig. S1 for remaining results).

For the sustained effects on appetite, decreased appetite expressed by summarised incremental AUC for VAS between time 0 and 240 min (before *ad libitum* lunch) was associated with lower energy intake at the *ad libitum* lunch (overall appetite suppression score: *R* −0·44, *P* = 0·003; satiety score: *R* −0·42, *P* = 0·006; fullness score: *R* −0·40, *P* = 0·048), whereas no associations were found for the acute effects on appetite (all *P* > 0·13) (see Supplementary Fig. S2).

Additionally, increased TFEQ restraint during the weight loss maintenance period was associated with improved weight loss maintenance (*R* −0·30; *P* = 0·027), whereas increased TFEQ disinhibition was associated with larger weight regain (*R* 0·32; *P* = 0·023). TFEQ hunger during the weight loss maintenance period was, however, not found to be associated with weight loss maintenance (*P* = 0·12).

### Relationship between appetite after weight loss and weight loss maintenance

Pre-maintenance VAS scores indicating a high level of appetite were not found to be associated with weight loss maintenance (all *P* > 0·22) (Table 3).

**Table 3.** Relationship between pre-maintenance visual analogue scale (VAS) scores indicating a high level of appetite and changes in body weight* (Unstandardised regression coefficients (β) and 95 % confidence intervals)

	Δ Weight (kg)			
iAUC pre-maintenance VAS scores indicating a high level of appetite (mm)	β	95 % CI	*R*	*P*
Hunger	−0·02	−0·04, 0·01	−0·30	0·22
Desire to eat	−0·01	−0·07, 0·01	−0·29	0·64
Prospective food consumption	−0·01	−0·03, 0·01	−0·31	0·37

iAUC, incremental AUC.

* Data are presented as unstandardised regression coefficients (β) and 95 % confidence intervals using linear mixed models including adjustment for age, sex, pre-maintenance body weight, body weight change during the low-energy diet period and baseline measure of appetite of interest (e.g. when examining association between iAUC baseline hunger and changes in body weight, the model was adjusted for baseline hunger at baseline) (fixed effects) as well as site (random effect).

Increased TFEQ disinhibition during the weight loss period was associated with weight regain during the weight loss maintenance period (*R* 0·33; *P* = 0·006). Changes in TFEQ restraint and hunger during the weight loss period were not found to be associated with weight loss maintenance (*P* = 0·28; *P* = 0·50). Pre-maintenance TFEQ eating behaviour characteristics were not found to be associated with weight loss maintenance (all *P* > 0·36; see Supplementary Table S1).

## Discussion

The results demonstrate that sustained reduction in 24-h energy intake from before to after the 12-week weight loss maintenance period explains 14 % of the variation in weight loss maintenance, whereas acute changes in 24-h energy intake were not found to be associated with weight loss maintenance. Suppression of self-reported appetite at both acute and sustained evaluations was associated with improved weight loss maintenance, explaining 11 and 10 % of the variation in weight loss maintenance, respectively.

### Relationship between acute *v.* sustained effects on appetite and body weight management

We only observed a minor weight regain with little variation, making the predictive value relatively low. Nevertheless, the large range in weight regain allows investigating the association between the individual values as done in previous studies. In previous studies by the groups of Martin^(22)^ and Wang^(23)^, decreased *ad libitum* energy intake after exposure to appetite-reducing interventions explained 58 % (*P* < 0·001) and 23 % (*P* < 0·001) of the variations in the subsequent weight losses over 12 and 8 weeks, respectively. The magnitudes and variations of the weight loss observed by the groups of Martin^(22)^ and Wang^(23)^ exceeded the weight regain that we observed. This could explain why appetite explained more of the variation in body weight in these studies compared with our results. Martin *et al*.^(22)^ also assessed differences in appetite by 24-h *ad libitum* energy intake, whereas Wang *et al*.^(23)^ assessed the relationship between differences in lunch *ad libitum* energy intake and weight loss. This indicates that products proven to decrease 24-h or lunch energy intake may be helpful in body weight management. This further indicates that 1-d assessments of appetite by *ad libitum* energy intake or self-reported evaluations may be indicators of habitual diet. Thereby, these assessments may be valuable markers of beneficial physiological effects on body weight management. Additionally, a particularly important feature of the present study is that a decreased snacking behaviour during afternoon/evening seems to be essential for body weight management, explaining 12 % of the variation in weight loss maintenance. This indicates that it is essential that new dietary-based concepts are able to reduce appetite during the entire day, and not only at meal times. Considering that snacking has been found to be positively associated with energy intake and that individuals with obesity have been found to be more frequent snackers than individuals of normal weight, it seems plausible that the ability to control snacking is important^(38)^. Additionally, effects on appetite obviously need to be sustained for a longer period to be beneficial in relation to body weight management. However, this does not necessarily mean that effects of products need to be demonstrated after repeated exposures during a long-term intervention. Halford *et al*.^(39)^ recently reviewed the sustained efficacy of products designed to reduce appetite when tested after acute and repeated exposures. They showed that acute robust satiety-enhancing and/or hunger-reducing effects are likely to be sustained. We were; however, unable to show a relationship between acute effects on energy intake and weight loss maintenance, whereas acute effects on self-reported appetite evaluations were found to be related to weight loss maintenance. In our study, the participants stopped the LED 1–2 weeks prior to the first and the second appetite probe days. Thus, we aimed to avoid that the participants were in a strong negative energy balance at the appetite probe days. Nevertheless, the participants only recently regained access to normal food after 8 weeks of low-energy formula diet. Thereby, it may have been difficult for the participants to control their cravings for food when exposed to the *ad libitum* meals despite being instructed only to eat until comfortably full, creating uncertainty about the acute effects on energy intake. This seems to be supported by our results showing no relationship between the self-reported appetite evaluations prior to the lunch and energy intake at the *ad libitum* lunch when acute effects on appetite were assessed. However, sustained effects on self-reported appetite evaluations were found to be related to following *ad libitum* energy intake. We thereby hypothesise that those reporting acute suppression of appetite when evaluated by VAS do experience reduction in appetite throughout the weight loss maintenance period, helping them to consume less during the weight loss maintenance period; thus, the satiating effect is beneficial for their weight loss maintenance. In general, suppression of appetite assessed by VAS is not necessarily translated into reduced energy intake at the following meals and results are not directly comparable^(40,41)^. Both measures are prone to self-reporting and social-desirability biases as well as being affected by potential variation in the instruction of the participants^(33,42)^. *Ad libitum* energy intake is believed to be a more direct measure of eating behaviour; however, this method may be affected by food cravings, liking of the meals served, etc.^(43)^. Self-reported appetite evaluations are affected more by day-to-day variation than *ad libitum* energy intake and subjective interpretations of the questions may also affect the answers^(44,45)^. Comparable with our results, Porrini *et al.*^(46)^ previously showed that self-reported evaluations of satiety and fullness do not completely overlap. Porrini *et al*.^(46)^ found that reports of fullness may be influenced by the kind of food eaten, possibly explaining why we found no relationship between changes in reports of fullness and weight loss maintenance. Also, interpretation of self-reported appetite evaluations and energy intake is not always obvious, as feeling of appetite is both an antecedent of energy intake and a consequence of having eaten.

### Relationship between changes in eating behaviour characteristics during weight loss maintenance and body weight management

We showed that increasing TFEQ restraint during weight loss maintenance is associated with improved weight loss maintenance. This is somewhat contradictory to previous studies showing that high pre-maintenance restraint was inversely associated with initial weight loss and furthermore linked with subsequent weight regain^(47,48)^. Thereby it is unclear whether restrained eating behaviour is helpful or detrimental regarding body weight management. It has been argued that restrained eating increases the spontaneous neural activity in food reward and inhibitory brain regions resulting in increased energy intake^(47,48)^. However, these studies assessed restrained eating behaviours using the Dutch restrained eating scale and the Dutch eating behaviour questionnaire. TFEQ restraint has been found not to be related to body weight changes and participants characterised as being highly restrained eaters based on the TFEQ have been found to have a lower energy intake than low-restraint eaters^(36,49,50)^. It has been argued that other questionnaires, like the Dutch restrained eating scale and the Dutch eating behaviour questionnaire perceived to assess restraint, are actually assessing what is characterised as disinhibition in the TFEQ^(36)^. This was confirmed by another study showing that no measure of impulsivity was related to TFEQ restraint, whereas TFEQ disinhibition was related to impulsivity and predicted the likelihood of developing overeating^(51)^. We thereby hypothesise that restrained eating in the sense of ability to control food intake may be beneficial to manage body weight. Additionally, we found that increased TFEQ disinhibition during weight loss maintenance was associated with a larger weight regain. Disinhibited eating behaviour, i.e. high responsiveness to food stimulating the onset of eating, has been shown to be related to hunger and impulsivity resulting in increased energy intake^(36,51,52)^. Thus, our results confirm that low disinhibited eating behaviour characteristic is important for maintaining weight loss.

### Relationship between appetite after weight loss and following weight regain

Self-reported appetite evaluations indicating a high level of appetite pre-maintenance were not found to be risk factors for weight regain. Reports of appetite during single days only provide an estimate of the level of appetite after the weight loss, but it remains unknown whether appetite has increased during the weight loss period or not^(43)^. Based on the previously shown changes in physiological signals known to stimulate appetite after weight loss^(14–16)^, it seems plausible to hypothesise that increased appetite is a risk factor for weight regain. Since no appetite probe day was included before the LED, we were unable to investigate this based on measures of appetite collected at the standardised appetite probe days. However, we found that increasing TFEQ disinhibition during weight loss was a risk factor for weight regain, whereas pre-maintenance TFEQ scores were not found to be association with weight regain. These results support that single measures after the weight loss do not provide sufficient information as the starting point seem to be important. To investigate this objective, changes during the weight loss are thereby more reliable, whereby it would have been beneficial to include an appetite probe day before the LED period. Nevertheless, the TFEQ represents an enduring effect on eating behaviour during the past weeks and thereby may be a reliable measure for the association we were interested in investigating rather than single measures from the appetite probe day after the initial weight loss period^(36,50)^. Disinhibition has been shown to be a reliable predictor for unwanted eating behaviours and to be closely related to food sensitivity or factors that influence the onset of eating^(52)^. Previous studies have also found a link between high TFEQ disinhibition and weight regain^(52–54)^. It thereby seems plausible that increased disinhibited eating behaviour during weight loss is a risk factor for weight regain. We also recently showed that increased TFEQ disinhibition and hunger during a weight-loss period was associated with less weight-loss success^(24)^. In the present paper, we showed that increased TFEQ disinhibition both during weight loss and during weight loss maintenance was associated with larger weight regain in the following weight loss maintenance period. Thus, increased appetite during weight loss is probably a risk factor for subsequent weight regain. In particular, individuals experiencing increased disinhibited eating behaviour may require additional support both during weight loss and weight loss maintenance in order to manage body weight.

### Strengths and limitations

High completion rate strengthens the conclusion, which is based on results from the majority of the participants initiating the weight loss maintenance period. Site differences may have occurred; however, no differences were found when checking the statistical models and potential differences were also accounted for by including site as a random effect in all statistical analyses. Both men and women were included in this study, representing the whole target population for products designed to reduce appetite. However, phase of menstrual cycle has been shown to affect appetite^(55–57)^, and this was not taken into account at the appetite probe days. It would have strengthened the results to only test women in the follicular phase, but this was deselected due to practical reasons of fitting appetite probe days and the associated study visits within the predetermined visit windows. Information on participants’ motives for participating in the study as well as previous dieting history was not included, which limits the ability to consider how representative the sample is. Nevertheless, volunteers are usually motivated to lose weight and have tried other strategies without success, whereby they seek this help. Thereby we assume that these results can also be generalised to chronic dieters. Allowing completion of the TFEQ at home was an advantage for the participants in regard to time constraints, and the participants were instructed to answer alone and try to avoid external disturbances. However, an uncontrolled environment while answering the TFEQ may have introduced increased variability.

### Conclusion

Reduction in appetite assessed by energy intake and self-reported appetite evaluations at standardised appetite probe days seems to be associated with improved body weight management. This indicates that reduced appetite may be a beneficial physiological effect on body weight management in the context of overweight and obesity. Food products able to reduce appetite may thereby be an interesting strategy for dietary-based treatments, expanding the ‘toolbox’ needed to help people manage body weight in order to maintain health and wellbeing throughout life. Self-reported evaluation indicating a high level of appetite after weight loss was not found to be a risk factor for weight regain, but it remains interesting to investigate whether increased appetite after weight loss may be a risk factor for weight regain.

## Supplementary material

For supplementary material accompanying this paper visit http://dx.doi.org/10.1017/jns.2019.36. click here to view supplementary material
