# Supplementary material for: Is reduction in appetite beneficial for body weight management in the context of overweight and obesity? Yes, according to the SATIN (Satiety Innovation) study
Source: J Nutr Sci. 2019 Nov 27;8:e39. doi: 10.1017/jns.2019.36 (PMC6984007; doi:10.1017/jns.2019.36)
Supplement: Supplementary file 1 [file S2048679019000363sup001.pdf]

Is reduction in appetite beneficial for body weight management in the context of overweight and obesity? Yes, according to the SATIN (Satiety Innovation) study

Supplementary material

Supplementary results on relationship between reduction in appetite and weight loss maintenance

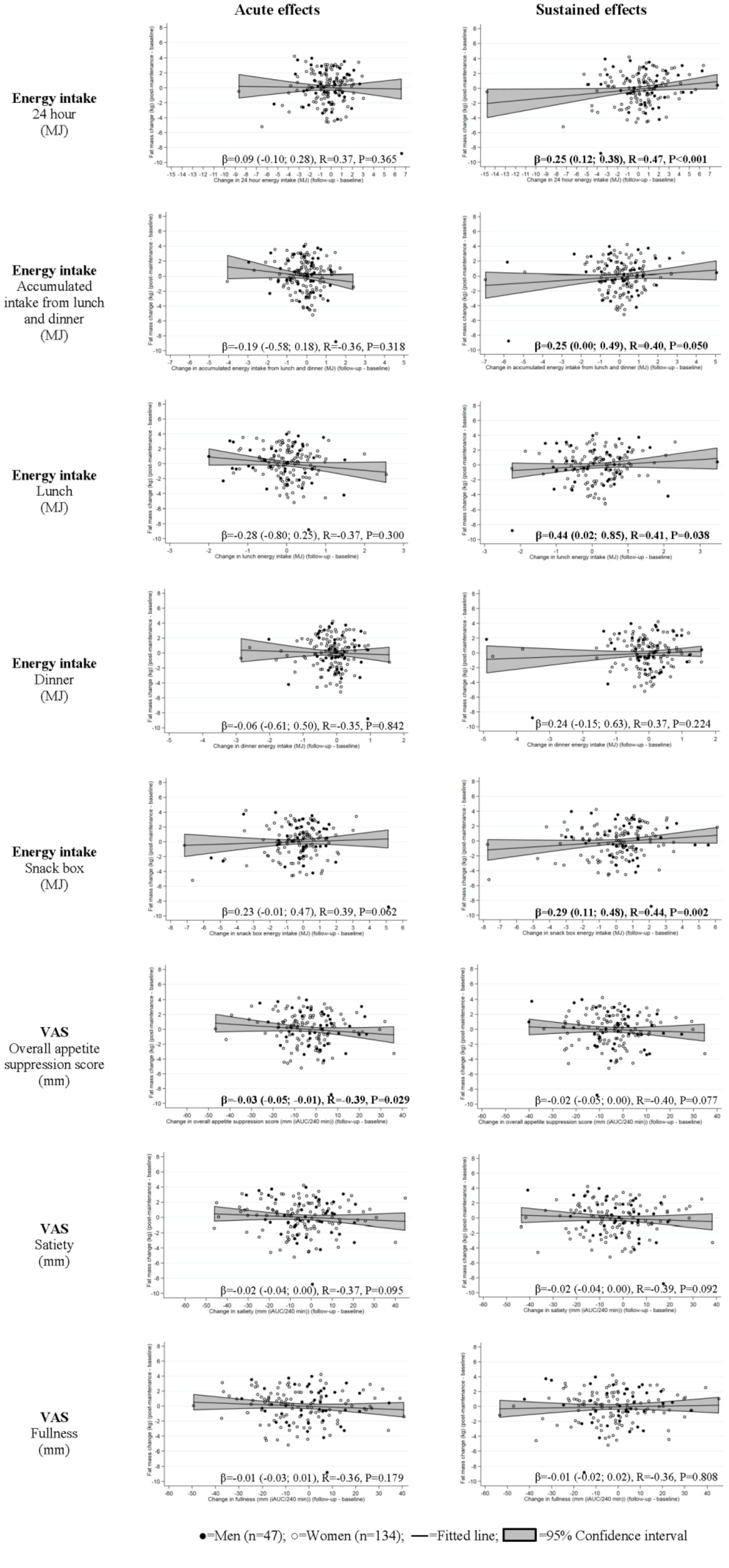

Figure 1 Relationship between changes in appetite (24-hour energy intake, energy intake at each of the *ad libitum* meals and summarised iAUC of each of the self-reported appetite evaluations divided by acute [after a single exposure] and sustained [after repeated exposures] effects) and changes in fat mass (kg) from pre- to post-maintenance. iAUC, Incremental area under the curve; VAS, Visual analogue scales, Acute effects, Difference in measures of appetite between the first and the second appetite probe days; Sustained effects, Difference in measures of appetite between the first and the third appetite probe days; Overall appetite suppression score = (satiety + fullness + [100-hunger] + [100-desire to eat] + [100-prospective food consumption]) / 5; 0 indicates higher appetite/less satiety and 100 indicates lower appetite/more satiety.

Positive change in energy intake equals increased energy intake/higher appetite. Positive change in self-reported appetite evaluation equals decreased appetite. Positive change in fat mass equals fat mass regain after the weight loss maintenance period.

Data are presented as unstandardised regression coefficients (β) and 95% confidence intervals and correlations coefficients using linear mixed models including adjustment for age, gender, pre-maintenance fat mass, fat mass change during the low energy diet period and baseline measure of appetite of interest (e.g. when examining association between changes in 24-hour energy intake and changes in body weight, the model was adjusted for 24-hour energy intake at baseline) (fixed effects) as well as site (random effect).

Is reduction in appetite beneficial for body weight management in the context of overweight and obesity? Yes, according to the SATIN (Satiety Innovation) study

Supplementary material

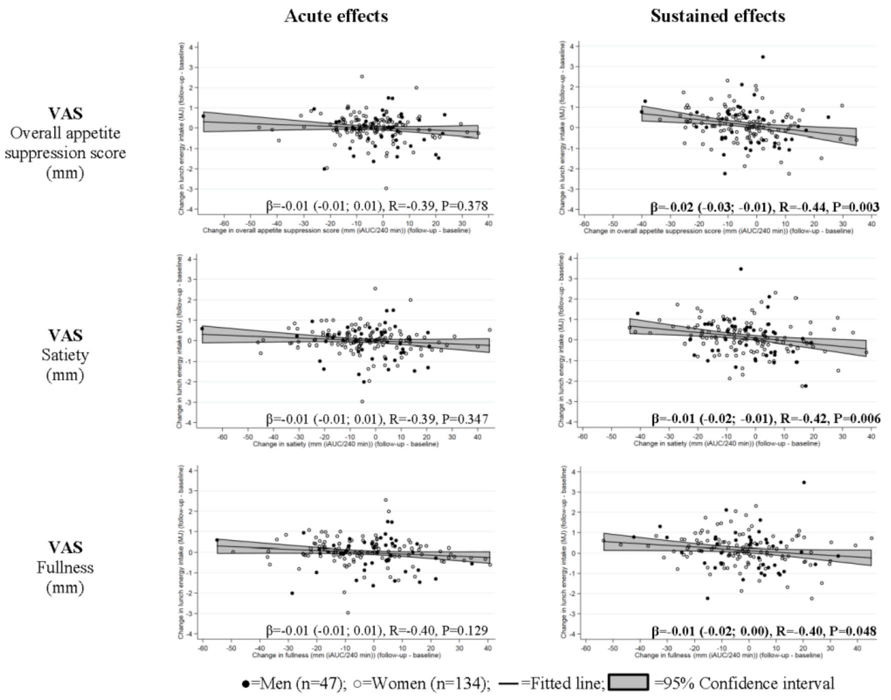

Figure 2 Relationship between changes in each of the self-reported appetite evaluations (summarised incremental area under the curve for VAS time 0-240 min [before *ad libitum* lunch], divided by acute [after a single exposure] and sustained [after repeated exposures] effects) and changes in *ad libitum* lunch energy intake.

iAUC, Incremental area under the curve; VAS, Visual analogue scales, Acute effects, Difference in measures of appetite between the first and the second appetite probe days; Sustained effects, Difference in measures of appetite between the first and the third appetite probe days; Overall appetite suppression score = (satiety + fullness + [100-hunger] + [100-desire to eat] + [100-prospective food consumption]) / 5; 0 indicates higher appetite/less satiety and 100 indicates lower appetite/more satiety.

Positive change in self-reported appetite evaluation equals decreased appetite. Positive change in energy intake equals increased energy intake/higher appetite.

Data are presented as unstandardised regression coefficients (β) and 95% confidence intervals and correlations coefficients using linear mixed models including adjustment for age, gender, baseline lunch energy intake and baseline measure of appetite of interest (e.g. when examining association between changes in iAUC overall appetite suppression score and changes in lunch energy intake, the model was adjusted for baseline appetite suppression score at baseline) (fixed effects) as well as site (random effect).

Is reduction in appetite beneficial for body weight management in the context of overweight and obesity? Yes, according to the SATIN (Satiety Innovation) study

Supplementary material

Supplementary results on relationship between appetite after weight loss and weight loss maintenance

Table 1 Relationship between pre-maintenance TFEQ eating behaviour characteristics and changes in body weight (mean [95% CI]).

Pre-maintenance TFEQ eating behaviour characteristic	ΔWeight (kg)	R	P-value
Restraint	-0.01 (-0.08; 0.08)	-0.24	0.98
Disinhibition	0.05 (-0.06; 0.16)	0.26	0.36
Hunger	0.03 (-0.08; 0.14)	0.24	0.56

TFEQ, Three-factor eating questionnaire; CI, Confidence interval.
Data are presented as unstandardised regression coefficients (β) and 95% confidence intervals and correlations coefficients using linear mixed model including adjustment for age, gender, pre-maintenance body weight and body weight change during the low energy diet period (fixed effects) as well as site (random effect).
